# Perspectives for integrated insect pest protection in oilseed rape breeding

**DOI:** 10.1007/s00122-022-04074-3

**Published:** 2022-03-16

**Authors:** Christian Obermeier, Annaliese S. Mason, Torsten Meiners, Georg Petschenka, Michael Rostás, Torsten Will, Benjamin Wittkop, Nadine Austel

**Affiliations:** 1 Department of Plant Breeding, Justus Liebig University, Heinrich-Buff-Ring 26-32, 35392 Giessen, Germany; 2 Plant Breeding Department, University of Bonn, Katzenburgweg 5, 53115 Bonn, Germany; 3 Institute for Ecological Chemistry, Plant Analysis and Stored Product Protection, Julius Kühn Institute, Koenigin-Luise-Str. 19, 14195 Berlin, Germany; 4 Department of Applied Entomology, University of Hohenheim, Otto-Sander-Straße 5, 70599 Stuttgart, Germany; 5 Division of Agricultural Entomology, University of Göttingen, Grisebachstr. 6, 37077 Göttingen, Germany; 6 Insitute for Resistance Research and Stress Tolerance, Julius Kühn Insitute, Erwin-Baur-Str. 27, 06484 Quedlinburg, Germany

## Abstract

In the past, breeding for incorporation of insect pest resistance or tolerance into cultivars for use in integrated pest management schemes in oilseed rape/canola (*Brassica napus*) production has hardly ever been approached. This has been largely due to the broad availability of insecticides and the complexity of dealing with high-throughput phenotyping of insect performance and plant damage parameters. However, recent changes in the political framework in many countries demand future sustainable crop protection which makes breeding approaches for crop protection as a measure for pest insect control attractive again. At the same time, new camera-based tracking technologies, new knowledge-based genomic technologies and new scientific insights into the ecology of insect–*Brassica* interactions are becoming available. Here we discuss and prioritise promising breeding strategies and direct and indirect breeding targets, and their time-perspective for future realisation in integrated insect pest protection of oilseed rape. In conclusion, researchers and oilseed rape breeders can nowadays benefit from an array of new technologies which in combination will accelerate the development of improved oilseed rape cultivars with multiple insect pest resistances/tolerances in the near future.

## Oilseed rape breeding and production within changing environments

Oilseed rape/canola (*Brassica napus*) is a very young cultivated species originating from interspecific hybridisation of its natural ancestors *B. oleracea* (cabbage) and *B. rapa* (turnip rape) a few thousand years ago through domestication and intensive artificial selection by humans (Chalhoub et al. 2014). Natural wild forms of *B. napus* are not known (Mason and Snowdon 2016). Oilseed rape is a major crop in central Europe, Canada, China, South Asia and Australia and has significant economic value (e.g. Neik et al. 2017). Moreover, oilseed rape plays an important role in the improvement in soil structure and disease suppression for cereal crops (Angus et al. 2015) and is thus a favourable preceding or break crop in rotation schemes in cereal-dominated agricultural production systems (e.g. Sieling and Christen 2015). Due to the strong and constant selection for some major traits (e.g. low erucic acid and glucosinolate seed content) and the extensive crossbreeding of cultivars in rapeseed breeding programs worldwide over the last 70 years, severe genetic bottlenecks were imposed and the genetic diversity of modern cultivars has been enormously reduced (Mason and Snowdon 2016). This loss of genetic diversity may also have been accompanied by a loss of defence compounds and resistance against fungal pathogens and insects in modern elite cultivars (Gols et al. 2008a; Chen et al. 2015). Oilseed rape is susceptible to many fungal diseases and insect pests (e.g. Hegedus and Erlandson 2012; Neik et al. 2017). A global survey among 22 experts from 10 countries on major biotic constrains of oilseed rape production in 2019 revealed 16 diseases and 37 insect pests, as well as nematodes, slugs and snails, to be present in oilseed rape or mustard since 2016 (Zheng et al. 2020). Globally, insect pests play a more important role than fungal diseases in oilseed rape production, except for Australia. With 17 insect pests, Europe has the highest diversity compared to China, Canada and Australia. Only one single insect, the diamondback moth (*Plutella xylostella*), has been reported to occur worldwide. Damage by insect pests is resulting in an annual yield loss in oilseed rape production of 15% on a European scale (Milovac et al. 2017). In contrast with the management of fungal diseases, management of insect pests in oilseed rape has relied on pesticides in the last decades due to the lack of effective methods of crop rotation, tillage, biocontrol and cultivar resistance (Zheng et al. 2020). The frequent use of a limited number of insecticides sharing the same mode of action resulted in insect populations with resistance against various classes of insecticides (e.g. pyrethroids) within the last decade worldwide including populations of the diamondback moth, of the pollen beetle (*Brassicogethes aeneus*), of the cabbage stem flea beetle (*Psylliodes chrysocephala*) and of the cabbage seed weevil (*Ceutorhynchus obstricus*, syn. *assimilis*) (Hervé 2018). In Europe, the ban of neonicotinoid seed treatments in 2013 resulted in an increased insect damage and decreased yield of oilseed rape production (e.g. in the UK, Dewar 2017). Also, new product approvals for conventional crop protection products are declining (Phillips McDougall 2018). At the same time, climate change is resulting in changes of insect populations and, consequently, movement of insect pests into so far non-affected oilseed rape production areas has been predicted (Bale et al. 2002). These developments are currently threatening oilseed rape cultivation. Because insect pest management strategies exclusively relying on insecticides are not sustainable, a fundamental shift towards innovative and integrated management approaches is urgently required.

## Challenges and chances for breeding towards effective insect resistance

For maize, rice and wheat, it has been reported that publicly funded crop breeding for insect resistance is a long story of success, and according to Smith (2021) conventional breeding of insect-resistant crop plants is still the best way to feed the world population. Known resistances against insects are predominantly based on quantitative resistances while qualitative, monogenic resistances based on gene-for-gene interactions are rare and limited to phloem-sucking insects (VanDoorn and de Vos 2013; Kliebenstein 2014). However, for these monogenic inherited insect resistances plant breeding companies have successfully exploited natural variation in the last decades in several horticultural and agricultural crops (VanDoorn and de Vos 2013). In contrast, no monogenic resistance against any insect pest in oilseed rape is known and currently no cultivars are available which show tolerance or resistance against any of the commercially important insect pests (Hervé 2018). Also, in the primary germplasm pool of older oilseed rape cultivars no reduced insect susceptibilities has been found—except for aphids (e.g. Dunn and Kempton 1969; Table 1).

**Table 1:** Literature summary for reduced susceptibility and resistance against insect pests recorded in (1) *B. napus*, (2) in its progenitor species and (3) in wider relatives

Insect pest	Reduced susceptibility in *B. napus*	Reduced susceptibility in *B. rapa*, *B. oleracea,* resynthesized *B. napus*	Resistance/tolerance in other brassicaceae species
Pollen beetle (*Brassicogethes aeneus*, *Meligethes aeneus*)	No (Åhman 1993; Hervé and Cortesero 2016; Hervé et al. 2014; Austel et al. 2021)	Some (Austel et al. 2021)	*S. alba*, *E. sativa*, *D. tenuifolia*, *B. vulgaris* (Hervé and Cortesero 2016; Austel et al. 2021)
Cabbage stem weevil (*Ceutorhynchus pallidactylus*)	Some (Eickermann and Ulber 2010)	Some (Eickermann et al. 2011)	
Rape stem weevil (*C. napi*)	No (Schaefer et al. 2017)	Some (Schaefer-Koesterke et al. 2017)	
Cabbage seedpod weevil (*C. obstricus*, syn. *C. assimilis*)	No (Åhman 1993)		*S. alba* (Ross et al. 2008; Tansey et al. 2010a; Lee et al. 2014)
Cabbage stem flea beetle (*Psylliodes chrysocephalus*)		Some (Döring and Ulber 2020)	*S. alba*, *R. sativa* (Döring and Ulber 2020)
Crucifer flea beetle (*Phyllotreta cruciferae*)	Some (Heath 2017)		*S. alba*, *T. arvense*, *C. sativa*, *Crambe* ssp. (Gavloski et al. 2000; Soroka and Grenkow 2013)
Striped flea beetle (*Pyllotetra striolata*)	Some (Heath 2017)		
Yellow-striped flea beetle (*Pyllotetra nemorum*)			*B. vulgaris* (Kuzina et al. 2011)
Diamondback moth (*Plutella xylostella*)		Some (Kim et al. 2013)	
Bertha armyworm (*Mamestra configurata*)			*T. arvense*, *M. sativa*, *S. alba*, *B. juncea* (Dosdall and Ulmer 2004; Ulmer et al. 2001 and 2002)
Cabbage moth (*M. brassicae*)		Some (Cartea et al. 2010; Carmona et al. 2011)	
Cabbage whitefly (*Aleyrodes proletella*)		Some (Pelgrom et al. 2015)	*B. villosa*, *B. incana*, *B. montana* (Pelgrom et al. 2015)
Brassica pod midge (*Dasineura brassicae*)	No (Åhman 1993)		
Cabbage root fly (*Delia radicum*)	No (Dosdall et al. 1994)	Yes (Dosdall et al. 2000; Santolamazza-Carbone et al. 2017)	*B. fruiticolosa*, *B. spinescens*, *B. villosa*, *B. montana*, *B. hilarionis*, *B. macrocarpa* (Shuhang et al. 2016)
Green peach aphid (*Myzus persicae*)	Some (Palmer 1953; Sarwar and Sattar 2013; Kordan et al. 2021)		
Cabbage aphid (*Brevicoryne brassicae*)	Some (Palmer 1953; Dunn and Kempton 1969, Ellis and Farrel 1995)		*B. fruiticolosa*, *B. spinescens* (Ellis and Farrell 1995)
Mustard aphid (*Lipaphis erysimi*)			*E. sativa*, *B. carinata*, *B. fruiticolosa* (Kumar and Sangha 2017; Kumar and Banga 2017)
Small and large white butterfly (*Pieris brassicae* and *P. rapae*)			*B. nigra* (Fatouros et al. 2014; Afentoulis et al. 2021)

Also see summaries in Hervé and Cortesero (2016), Hervé (2018) andQuezada-Martinez et al. (2021)

Another reason why oilseed rape breeding companies and research institutions did not get engaged in insect resistance breeding in the past is the phenotyping bottleneck which prevented screening of hundreds of accessions in parallel (Goggin et al. 2015). Phenotyping for insect resistance is challenging because (i) major insect pest species are difficult to rear in the laboratory, (ii) plant–insect interactions depend strongly on the plant developmental stage and on the environment and (iii) phenotyping in the field relies on natural insect populations infesting the fields, which vary strongly between years and locations. However, in the last years methods have been developed that try to overcome this bottleneck and might enable more efficient insect resistance breeding by screening large plant populations of oilseed rape and other crops more rapidly and precisely. The development and application of low-cost high-throughput phenotyping (HTP) methods in combination with newly generated large genomics datasets are of critical importance for the future success of breeding insect-resistant oilseed rape cultivars. For plant diseases and insect pests, the application potential application of sensors in HTP has been rated low compared to other crop phenotyping traits under field conditions (Jin et al. 2020). The direct measurement of insect activity in the field by sensor-based HTP methods is challenging. Thus, for application in commercial breeding or pre-breeding indirect traits have to be identified which are simple and cost-effective to measure in high-throughput in the greenhouse or field and which are strongly correlated with the complex target traits mediating insect resistance and co-vary with insect performance (Goggin et al. 2015). These proxy traits should preferably be less susceptible to genotype × environment (G × E) interactions than insect performance, e.g. leaf surface temperature, photosynthetic activity and other physiological characteristics of crops will change under insect infestation and are associated with specific spectral features. For this reason, unmanned aerial vehicle phenotyping platforms have been considered a good choice for developing HTP platforms for insect resistance screening in the field in the future (Song et al. 2021b). Kloth et al. (2015) demonstrated that under controlled conditions high-throughput phenotyping for aphid feeding on leaf discs by an automated video tracking system is feasible using 344 *A. thaliana* accessions. Chen et al. (2012) screened large Arabidopsis populations by an indirect approach, using ELISA to detect *Turnip yellows virus* (TuYV) transmission, and used virus titers as a proxy trait for possible aphid resistance. This approach can be easily transferred to oilseed rape. Also, Kirkeby et al. (2021) showed that camera-based recognition of insect species affecting oilseed rape by machine learning approaches can be applied and might in the near future allow local spraying on field sections and local automated counting of insect species for evaluating insect genotype preferences on a plot basis in the field. The emerging high-throughput phenotyping technologies together with knowledge-based identification of breeding targets from fundamental studies on molecular/metabolomic networks (trait discovery) are also critical to enable us to breed for integrated insect pest protection by targeting and screening for plant–insect–antagonist interactions in the greenhouse and field. However, simplified and indirect approaches, as presented by Chen et al. (2012), carry the risk of not being able to identify some resistant genotypes, e.g. phloem-localised resistance mechanisms do not necessarily prevent an infection with TuYV.

Although breeding and host plant resistance always have been considered to be a key step in integrated pest management (IPM), it has been rarely addressed in the framework of biocontrol or biopesticides. IPM has mainly been dominated by individual isolated measures that have been used as replacements for chemicals, but not based on true integration of different management strategies (Thomas 1999; Deguine et al. 2021). Nevertheless, breeding seems to be the most promising technology within IPM, besides crop rotation. Therefore, breeding for insect resistance should be developed as a central part of an integrated protection strategy against insect pests in the future. To implement oilseed rape cultivars with a high resilience to combined biotic and abiotic stresses within IPM regimes, cultivar mixtures, diversified cultivars and intercropping have been suggested (Lamichhane et al. 2018). Synthetic cultivars are 1 type of diversified cultivars, which are produced based on random mating of a number of selected inbred lines with a superior general combining ability and subsequent propagation of bulked seed (Becker 1988). The cultivation of these heterogeneous synthetic cultivars might result in local adaptation, buffering across certain environments, e.g. by providing resilience against diverse insect pests, reduction in pest damage and stabilisation of yield—however, providing only a reduced yield level compared to modern homogeneous hybrid cultivars. For oilseed rape, the cultivation of synthetic cultivars is underexplored (Falk et al. 1998; Niemelä et al 2006), but might be one long-term strategy, e.g. by producing synthetic cultivars from founder parents with different glucosinolate profiles providing resistances against different generalist and specialist insect pest species of oilseed rape.

**Fig. 1:**
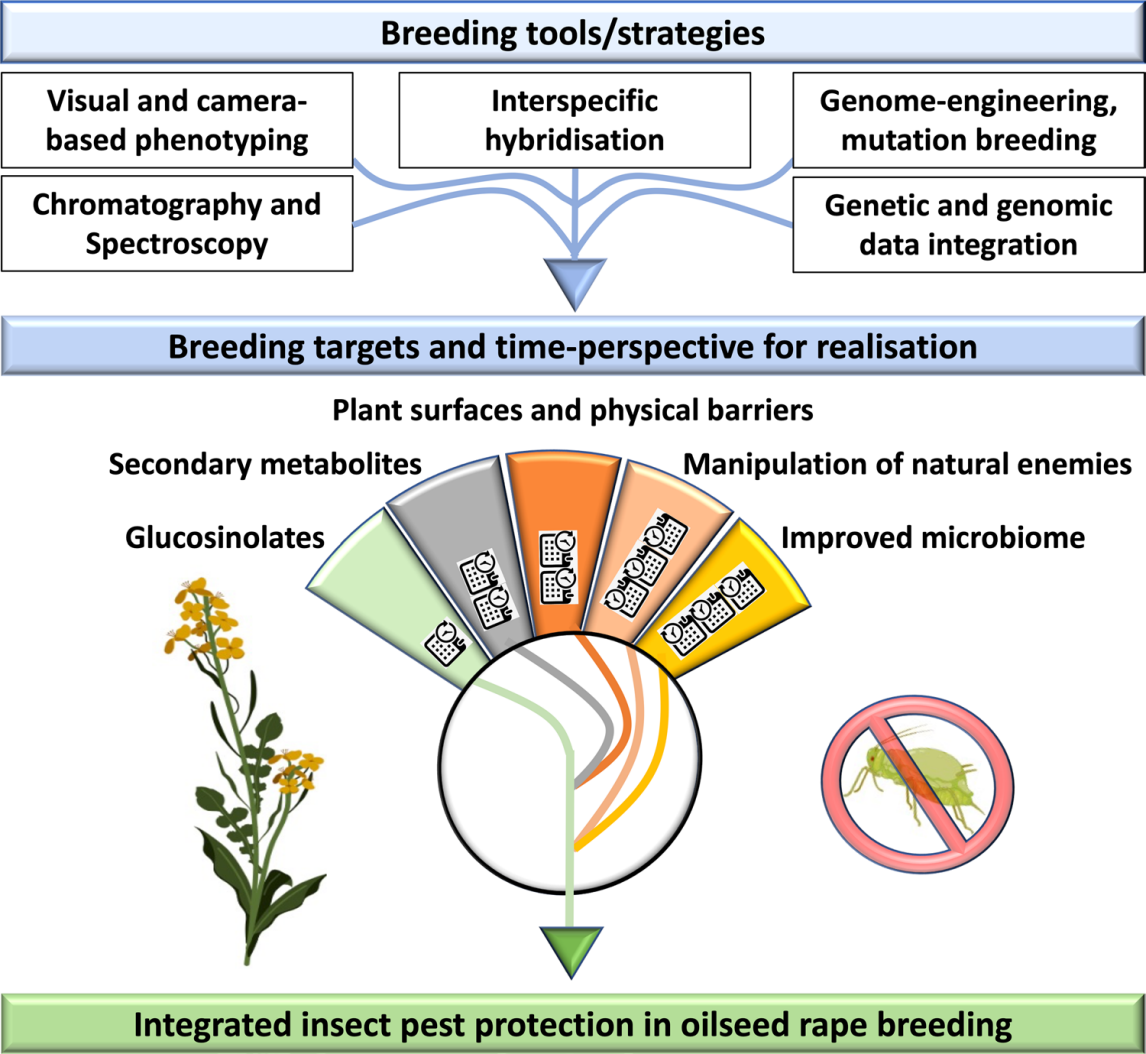
Schematic representation of major breeding tools/strategies and breeding targets with time-perspective for future realisation and availability of insect resistant breeding lines in integrated insect pest protection of oilseed rape.  Short-time perspective (5–15 years),  medium-time perspective (15–25 years),  long-time perspective (more than 25 years). Pictures are obtained from BioRender.com

Rapidly developing high-throughput phenotyping methods together with the increasing availability of resequencing and other genomic data for many *B. napus* lines, resynthesised *B. napu*s, and interspecific and intergeneric *Brassica* crosses open up new perspectives have recently emerged (Hu et al. 2021). The integration of genetic and genomic data to complement phenotyping data and the use of predictive algorithms in breeding is a process sometimes also referred to as Breeding 3.0. Soon, we will be entering a new phase in breeding for many crops including oilseed rape, termed Breeding 4.0. This breeding phase, which will be catalysed by major technical advances in high-throughput phenotyping, genetics and information systems, represents a new chance to develop insect resistant oilseed rape cultivars (Wallace et al. 2018). In the following parts (i) promising breeding resources and breeding strategies and (ii) target traits conferring insect resistance are discussed. The identified target traits have been classified based on their level of technological maturity and the time perspectives for realisation in breeding. Time perspectives for insect resistant breeding lines to be available for plant breeding companies addressing different breeding targets have been classified as short term with 5–15 years, medium term with 15–25 years and long term with more than 25 years (Table 2, Fig. 1).

**Table 2:** Overview on breeding targets, time-perspective for future realisation/availability of insect resistant breeding lines and recent bottlenecks for breeding towards insect resistance in oilseed rape (also see Fig. 1)

Direct and indirect breeding targets	Time-perspective for realisation	Breeding bottlenecks	Available new HTP methods	Most promising breeding targets suitable for HTP	References
Co-varying glucosinolates	Short-term	Phenotyping is HPLC based	NIRS	High overall GSL, see Table 3 for details	Font et al. (2005); Beckmann et al. (2007); Toledo-Martín et al. (2017)
Co-varying secondary metabolites	Medium to long-term	Phenotyping is mostly HPLC/GC based, lack of monofunctional metabolites	Chromatography	Terpenoids, alkaloids, phenolics	Boulogne et al. (2012); Lebot et al. (2021)
Plant surfaces and physical barriers	Medium-term	Visual phenotyping is low throughput	Camera-based leaf disc assessment, enzyme assay (virus titer as proxy for aphid resistance)	Trichomes for crucifer flea beetle, diamondback moth, aphids	Kloth et al. (2015); Chen et al. (2012); Alahakoon et al. (2016b); Mei et al. (2017); Divilov et al. (2017)
Manipulation of natural enemies: volatiles, plant-derived semiochemicals, insect repellents, attractants	Long-term	Phenotyping is mostly HPLC/GC based, lack of relevant field data, understanding	Camera-based laboratory tracking	Alkenyl glucosinolates, isothiocyanates, nitriles	Mauchline et al. (2018); Tansey et al. (2010a); de Bruijn et al. (2018)
Manipulation of natural enemies: tritrophic interactions, antagonist reinforcement	Long-term	Visual phenotyping is low throughput, lack of relevant field data, understanding	Multi-camera field monitoring	Unknown	de Bruijn et al. (2021)
Microbiome improvement	Long-term	Phenotyping, lack of field studies, lack of genetic studies for plant populations, lack of functional understanding	16S rRNA sequencing	Antagonistic, growth-promoting effects	Rochefort et al. (2021)

Short-term = 5–15 years, medium-term = 15–25 years, long-term = more than 25 years

## Breeding resources and breeding strategies

### Novel resistances from interspecific hybridisation

The primary germplasm pool of *B. napus* is thought to lack resistances to many insect pests (Hervé 2018 and summarised in Table 1). However, interspecific hybridisation has historically been widely used in the *Brassica* genus to introgress useful traits into crops (reviewed by Katche et al. 2019; Quezada-Martinez et al. 2021). Therefore, moving useful insect resistances from related species into *B. napus* via resynthesis and interspecific hybridisation is an approach which should be considered to address this problem. *B. napus* as an allotetraploid species with the A and C genomes shares a subgenome with progenitor species *B. rapa* (2*n* = AA) and *B. oleracea* (2*n* = CC), as well as with its sister allopolyploid species Indian mustard (*B. juncea*; 2*n* = AABB) and Ethiopian mustard (*B. carinata*; 2*n* = BBCC). Interspecific hybridisation to produce hybrids between *B. napus* and each of these species is relatively straightforward and has been achieved by hand pollination in each case without the need for embryo rescue or other interventions (reviewed by FitzJohn et al. 2007). Although crossing success is highly genotype dependent, the easiest cross is considered to be *B. napus* × *B. rapa*, followed by crosses with *B. juncea*, then *B. carinata* and finally *B. oleracea*. An additional major advantage of hybridisation with species which share a subgenome with *B. napus* is that resistances can be readily transferred via homologous recombination between chromosomes from the shared subgenome in the interspecific hybrid (reviewed by Mason and Chevre 2016). Hypothetically, even quantitative resistances could be transferred via A–A chromosome pairing from *B. rapa* and *B. juncea* to *B. napus* and via C–C chromosome pairing from *B. oleracea* and *B. carinata* to *B. napus*. The recombination between shared subgenomes approach is by far the easiest way to use interspecific hybridisation for rapeseed crop improvement and has been successfully used in the past for a number of traits (see Katche et al. 2019; Quezada-Martinez et al. 2021 for review). Unfortunately, these genomes are also less likely to contain useful resistances that are not present in rapeseed already than more distantly related species, due to the high level of gene conservation between the *Brassica* subgenomes present in the different species (Chalhoub et al. 2014). So far, some evidence suggests this also applies to insect resistance traits (Austel et al. 2021). Thus, we may need to also consider the wider relatives when breeding for insect resistance (Table 1).

Although there are many species which can hybridise with *B. napus* and for which we know there is at least a chance of chromosome introgression, there are several major barriers which need to be considered. Firstly, production of interspecific hybrids in the first place may require tissue culture intervention (rescue of fertilised ovules or embryos), or more exotic techniques such as somatic fusion (for very distantly related species, where sexual reproduction between species is not possible). Secondly, interspecific hybrids produced may only very rarely undergo recombination between subgenomes during meiosis (e.g. Gaebelein et al. 2019). This recombination is critical to transfer small chromosome segments containing the desired introgression locus from the wild relative to the crop. Transfer of a whole chromosome and subsequent production of disomic addition lines is generally undesirable due to linkage drag, i.e. the relatively high chance that many agronomically deleterious alleles or genes will also be transferred into the crop with the addition of this chromosome. However, recombination frequency is dictated both by sequence similarity (which generally decreases with increasing phylogenetic distance) and by a number of as-yet-unknown or not well-characterised genetic factors which may be specific to species or lineages (reviewed by Pelé et al. 2018; Mason and Wendel 2020). Therefore, recombination may either take place extremely infrequently, such that recovery of introgressed segments is a gamble and/or requires huge populations, or there may be little to no chance of recombination occurring between the target region containing the “wild” locus of interest and *B. napus*, due to poor homoeology or recombination rates in the target area (see e.g. Adamczyk-Chauvat et al. 2017). Despite these challenges, there are some examples of the use of wild relatives which do not have a shared genome for *Brassica* crop improvement (reviewed by Katche et al. 2019). For example, resistance to mustard aphid (*Lipaphis erysimi*) was recently successfully introgressed from wild species *B. fruticulosa* (F genome) into *B. juncea* (2*n* = AABB) (Agrawal et al. 2021). Furthermore, introgression of *Sinapis alba* into *B. napus* seems to be a promising strategy, as intergeneric hybrids were resistant to seed pod weevil (*Ceutorhynchus obstrictus*) and root maggots (*Delia radicum, Delia floralis*) (Dosdall et al. 2000; Dosdall and Kott 2006). However, due to the phylogenetical distance, this is a labour and time-intensive approach with a long-term perspective to gain stable hybrid *B. napus* lines.

An additional barrier to the use of interspecific hybridisation for improvement in insect resistance in *B. napus* is that we know very little about the underlying genetics or physiology of the insect resistance that has been identified in wild relatives. This is not 100% prohibitive, since we can carry out interspecific hybridisation without a good understanding of the genetics underlying a specific trait, as plant breeders have done for the last hundred or more years. However, a better understanding of the inheritance of the resistance and the utility in the field will help targeting traits for which interspecific hybridisation is a good choice of method, i.e. traits which are highly heritable, and which confer qualitative, monogenic (single locus) resistances with little environmental variance. Interspecific hybridisation is a breeding strategy which can be realised within a short term (use of resynthesised lines) to long-term range (secondary and tertiary germplasm) (Fig. 1). Wide *Brassica* relatives of *B. napus* show high potential for use (Table 1), but both prevalence of resistance and mechanisms of resistance in wild relatives are still very under-studied.

### Transgenic, genome engineering and mutation breeding approaches

In the past, transgenesis has been broadly applied to protect some major crop species against coleopteran and lepidopteran insect pests. Genes for endotoxins (Cry toxins) derived from the bacterium *Bacillus thuringiensis* (Bt) have been used commercially in transgenic crops (genetically modified organisms, GMOs) since the mid-1990s, most commonly in maize and cotton, but not in oilseed rape (Sanahuja et al. 2011). Although the first Bt oilseed rape lines, effective in the control of the diamondback moth and the cabbage looper (*Trichoplusia ni*), were developed at the same time when most other Bt crops were obtained (Hervé 2018), Bt oilseed rape lines were not marketed until today. However, Bt oilseed rape has been found to be effective also in field experiments against the diamondback moth and the corn earworm *Helicoverpa zea* (Ramachandran et al. 1998). One well-known problem of transgenesis is that in Bt crops the development of insect populations with resistance against Bt toxins is a common phenomenon. Populations of *P. xylostella* have been shown to develop resistance against microbial Bt formulations, and populations of Cry1A-resistant *P. xylostella* have been shown to survive on transgenic crucifers including oilseed rape (Sayyed et al. 2003). Other transgenes used in oilseed rape to create insect resistant plants were protease inhibitors, lectins and chitinases which showed varying effectivity (summarised in Hervé 2018).

Recent developments that focus on the application of agrobiotechnological approaches focus on RNA interference (RNAi), a cell-based mechanism leading to a strong silencing of gene expression (knock down) of a selected gene in a target organism. This mechanism can be exploited for crop protection by delivery of double stranded (ds) RNA to crop plants via spraying (spray induced gene silencing, SIGS) and via expression of ds RNA coding constructs in transgenic plants (Zotti et al. 2018; Liu et al. 2020a, b; Chung et al. 2021). The agrobiotechnological approach that uses RNAi technology mediated by dsRNA expressing transgenic plants is also termed host-induced gene silencing. However, although many studies showed insect growth inhibition as well as an increasing lethality as a consequence of dsRNA feeding for many insect pest species, indicating a high potential of this approach for efficient insect pest control, only a few of these approaches have been transferred into commercial products as genetically engineered plants and none as commercially available spray applications. This suggests that more research into the efficient delivery, reduced degradation and reduced off-target effects in beneficial species is required (Chung et al. 2021).

More recently, powerful highly specific genome engineering (or genome-editing, GE) technologies, especially CRISPR/Cas9-based gene editing, have become available and have been applied to manipulate genes in oilseed rape increasing the toolbox for agrobiotechnology. In contrast with RNAi-based approaches which are leading to a knock down of expression GE approaches can lead to a complete knock out of the expression of selected genes. These GE technologies might have a promising perspective for application in molecular breeding of oilseed rape (summarised in Chang et al. 2021; Gocal 2021). Many examples for different plant species exist where genome editing was used to induce plant resistance against fungal pathogens, e.g. *Sclerotinia sclerotiorum* in oilseed rape (Sun et al. 2018), powdery mildew in tomato (Nekrasov et al. 2017), *Magnaporthe oryzae* in rice (Wang et al. 2016) and *Phytophthora infestans* in potato (Kieu et al. 2021). In all these examples, different susceptibility genes, e.g. *mlo* genes (Nekrasov et al. 2017), were knocked out. Research and breeding in crops including oilseed rape have been focusing for a long time on plant resistance genes (R genes), e.g. for development of improved oilseed rape cultivars with resistance against fungal pathogens (Lv et al. 2020). However, single dominant R genes are often overcome in the field by pathogens’ evolution within a few seasons (Rouxel and Balesdent 2017). Enhancing natural immunity by understanding and implementing ‘susceptibility’ gene-mediated natural pathogen and insect resistance mechanism by CRISPR/Cas9-based knockout is an attractive alternative to the nowadays commonly applied time-consuming breeding for R gene-mediated resistance (Pavan et al. 2010; Zaidi et al. 2018). ‘Susceptibility’ genes are coding for plant factors which are targeted by pathogen/pest effector proteins controlling plant susceptibility. Insect proteins can be found inside the saliva/oral secretions, which, for example, function as effector proteins suppressing plant defence responses. Most insect effectors were identified for aphids in Arabidopsis so far, e.g. for the green peach aphid (*M. persicae*) (Bos et al. 2010; Elzinga et al. 2014; Mugford et al. 2016; Wang et al. 2021). Only rarely the function of effectors has been elucidated, often because knock down of ‘susceptibility’ genes only provides quantitative effects and a slight decrease in susceptibility (Åhman et al. 2019). GE is still an emerging tool and for insect resistance there are only a few examples where GE was applied to plants making them resistant against economically important insect pests (e.g.Tyagi et al. 2020). CRISPR/Cas9 holds large promise as a breeding tool for specific and fast editing of target genes. In cases where suitable target genes have been identified, it has a short-term perspective for realisation in engineering insect resistance in oilseed rape. In most cases, more research is required to identify the most suitable target genes exhibiting strong effects on insect resistance of oilseed rape in the field also considering the polyploid nature of the crop (Mason and Snowdon 2016). Although GE by CRISPR/Cas9 is suggested to have a high economic potential as a new breeding technique, its commercial exploitation in breeding is financially risky and unattractive for companies in the near future due to current regulations by process-based criteria in the European Union according to the rules for genetically modified organisms (GMOs). This is not the case in many other countries like the USA, Argentina, Canada and China where GE is regulated by product-based criteria or on a case-by-case basis (Zhang et al. 2020).

Progress in random mutagenesis approaches like TILLING (Targeting-Induced Local Lesions in Genomes) allowing the fast detection of mutations in any genome has made this technology an alternative to the targeted CRISPR/Cas9 mutation technology, especially in Europe, as TILLING is not covered here under GMO legislation rules (Holme et al. 2019). Also, for *B. napus* substantial genomic and pan-genomic resources are nowadays available (e.g. Song et al. 2021a) which speed up analysis of the available *B. napus* TILLING populations for detection of mutations in target genes. This makes mutant *B. napus* populations (Wang et al. 2008; Harloff et al. 2012; Gilchrist et al. 2013) a valuable resource for reverse genetic approaches, e.g. to identify variation in glucosinolate genes useful for breeding of oilseed rape with modified glucosinolate profiles and insect resistance (see "Breeding for modified glucosinolate profiles in non-seed plant organs" section). New opportunities for the identification of useful mutants are also arising particular due to the availability of more powerful mutant population analysis methods based on the application of high-throughput amplicon or whole genome sequencing approaches designated TILLING by sequencing (e.g. Sashidhar et al. 2019).

The improvement in specificity and throughput of genome editing and genome-wide mutation analysis technologies achieved in recent years makes the introduction of new insect resistance traits into oilseed rape via these technologies a breeding strategy with a promising short-term perspective (except for Europe for GE due to legalisations issues, Fig. 1).

In the following sections, we are presenting promising breeding targets based on the current knowledge on their involvement in insect resistance/tolerance in oilseed rape.

## Breeding targets conferring insect resistance

### Breeding for modified glucosinolate profiles in non-seed plant organs

Glucosinolates (GSL) are involved in the defence of plants against pathogens and herbivorous insect (Ahuja et al. 2010; Liu et al. 2021). Brassicaceae species react upon insect herbivory with a “mustard oil bomb” consisting of GSL and their hydrolyzing enzymes (myrosinases) to release toxic degradation products that act as insect deterrents and toxins (Matile 1980; Chhajed et al. 2020). Glucosinolates, their breakdown products and plant volatile compounds (see below) hold great prospects for application in integrated pest management (Ahuja et al. 2010). However, some specialist insects have developed metabolic countermeasures to handle this “mustard oil bomb” and are even attracted by certain GSL and their hydrolytic products as cues for oviposition and feeding (Giamoustaris and Mithen 1995 ; Björkman et al. 2011). Oilseed rape has undergone severe genetic bottlenecks in the 1970s and 1980s due to intensive breeding for seed nutritional quality improvement for cultivars with low levels of seed GSL and low levels of seed erucic acid (00 cultivars). Selection for low total seed GSL content is performed as a standard selection approach on a regular basis by commercial breeders using non-destructive near infrared-reflectance spectroscopy (NIRS) technology (Hom et al. 2007). Besides the reported general positive correlation of seed and leaf GSL concentrations, low seed GSL (00) oilseed rape cultivars tend to contain similar concentrations of GSL in the leaves compared to high seed GSL (0) oilseed rape cultivars suggesting that breeding for the low seed GSL trait did not indirectly select for reduced total leaf GSL content or modified leaf GSL profiles in *B. napus* (Schilling and Friedt 1991; Mithen et al. 1992; Beckmann et al. 2007; Liu et al. 2020a, b). In addition, 00 cultivars have been shown to exhibit similar susceptibilities to pests and pathogens compared to 0 cultivars (Mithen 1992). Some studies in *A. thaliana* and *B. napus* also suggest that GSL produced in seeds and vegetative tissues are regulated independently (Fieldsend and Milford 1994; Li et al. 1999; Rosa 1997; Brown et al. 2003). Recently, Liu et al. (2020a, b) identified a candidate gene on chromosome A03 responsible for high leaf and low seed GSL concentrations and reported an independent inheritance of seed and leaf GSL in *B. napus*. Moreover, this candidate gene explains about 30% of the leaf GSL variation in low seed GSL genotypes and seems not to be fixed during the selection in double-low oilseed rape breeding programs (Liu et al. 2020a, b). Tan et al. (2021) recommended the mining for further alleles involved in the transport of GSL for the breeding of *B. napus* cultivars possessing low seed GSL and high GSL concentrations in vegetative tissues and identified *BnaC02.GTR2* as another potential candidate for altering seed and leaf GSL profiles. However, between resynthesised oilseed rape lines the total as well as individual GSL concentrations in the leaves differ substantially (Cleemput and Becker 2012). It was also found that these differences in leaves and stems of resynthesised *B. napus* are associated with reduced susceptibility against the cabbage stem weevil (*Ceutorhynchus pallidactylus*) and the rape stem weevil (*Ceutorhynchus napi*) (Eickermann et al. 2011; Schaefer-Koesterke et al. 2017; Schaefer et al. 2017). Similarly, it was found that in interspecific crosses of *B. napus* with *S. alba* the modified GSL profiles were associated with resistance to the cabbage seedpod weevil (McCaffrey et al. 1999; Dosdall and Kott 2006). Yet, breeding for modified GSL levels in vegetative tissues of oilseed rape has been described as a “double-edged sword.” Depending on the individual GSL compositions and concentrations, certain GSL might increase resistance to generalist pest herbivores, whereas they might at the same time also make plants more susceptible to specialist pest herbivores (Björkman et al. 2011; Bruce 2014; Hopkins et al. 2009). Thus, the nature of the GSL components addressed as indirect breeding targets for increasing insect resistance must be carefully chosen. Although some insects have developed strategies to overcome the toxicity of the glucosinolate–myrosinase system (Winde and Wittstock 2011), high concentrations of total GSL can affect even specialist insects and this may result in plant resistance (Hopkins et al. 2009; Björkman et al. 2011). GSL levels can be induced by herbivore damage, mainly by chewing insects which increase jasmonic acid levels in the plant, having positive or negative effects on subsequent herbivory (Bartlet et al. 1999; Ponzio et al. 2017; Badenes-Pérez 2021). As many glucosinolates and isothiocyanates are released upon tissue damage by the glucosinolate–myrosinase system chewing insects are more affected compared to phloem-sucking insects. Phloem-sucking insects are able to avoid most contact with myrosinase by feeding from the phloem (Sun et al. 2020), and some of them even sequester glucosinolates to use them against their enemies like the specialised cabbage aphid (*Brevicoryne brassicae*) (Kazana et al. 2007). However, non-specialised aphid species like the green peach aphid *M. persicae* are affected by an increase in indol GSL levels in leaves of *A. thaliana* (Kim et al. 2008). Elevated and reduced concentrations of total, isothiocyanate releasing and specific GSL in vegetative tissue of oilseed rape have been identified as suitable breeding targets for resistance against different generalist and specialist insect pest (summarised in Table 3). Mithen (1992) suggested to increase the overall leaf GSL levels without changing the relative proportions of different GSLs, and to increase concentrations of gluconapin, glucobrassicanapin and sinigrin, which are hydrolysed to isothiocyanates with high bioactivity against most tissue-damage causing pathogens and generalist insect pests. We suggest to breed for a decrease in glucobrassicin and neoglucobrassicin to provide resistance against weevils and root flies, to breed for a decrease in gluconasturtiin and propenyl isothyocyanates to provide resistance against weevils, to breed for a decrease in progoitrin to provide resistance against flea beetles and aphids and to breed for an increase in total GSL to provide resistance against aphids in *B. napus* elite cultivars (Table 3). Due to the high diversity of pest insects, there will be no particular glucosinolate compounds, which are active against all pest insects.

**Table 3:** Summary of reported effects and correlations of GSL groups and individual GSLs and breakdown products with resistance/reduced susceptibility in Brassicaceae species

Insect pest		Metabolome–insect association	Direct influence of single compounds on insect performance/behaviour	Reference(s)
Taxon	Species	Reduced susceptibility correlated with	negative–neutral–positive	
Coleoptera/nitidulidae	Broad spectrum insects (and fungi)	Increased: overall GSL, glucobrassicanapin, gluconapin, sinigrin Decreased: none		Mithen (1992; and references therein)
	Pollen beetle (*Brassicogethes aeneus* syn. *Meligethes aeneus*)	Increased: glucobarbarin, glucotropaeolin, dimeric glucosativin, sinalbin, sulforaphane Decreased: propenyl isothyocyanates	Glucobarbarin—negative Glucotropaeolin—neutral Sinalbin—neutral 2-phenylethyl isothiocyanate—positive Propenyl isothiocyanates—positive 5-hexenenitrile—positive 3-phenylpropionitrile—positive Indole—positive	Mithen (1992; and references therein); Smart and Blight (2000); Austel et al. (2021)
Coleoptera/curculionidae	Cabbage stem weevil (*Ceutorhynchus pallidactylus*)	Increased: 4-hydroxy-glucobrassicin Decreased: glucobrassicin, 4-methoxy-glucobrassicin, gluconasturtiin	2-phenylethyl isothiocyanate—positive	Eickermann et al. (2011); Walczak et al. (1998)
	Rape stem weevil (*C. napi*)	Increased: none Decreased: glucoalyssin, gluconasturtiin, glucobrassicanapin, glucobrassicin & neoglucobrassicin	2-phenylethyl isothiocyanate—positive	Schaefer-Koesterke et al. (2017); Schaefer et al. (2017); Walczak et al. (1998)
	Cabbage seedpod weevil (*C. obstricus*, syn. *C. assimilis*)	Increased: neoglucobrassicin, glucosinalbin, gluconapin Decreased: gluconasturtiin, neoglucobrassicin	3-butenyl isothiocyanate—positive 4-pentenyl isothiocyanate—positive 2-phenylethyl isothiocyanate—positive	Ulmer and Dosdall (2006); Evans and Allen-Williams (1998); Tansey et al. (2010a and 2010b)
Coleoptera/alticini	Cabbage stem flea beetle (*Psylliodes chrysocephalus* syn. *P. chrysocephala*)	Increased: none Decreased: progoitrin, 4-hydroxy-glucobrassicin, none	Sinigrin—neutral Glucobrassicin—positive Gluconapin—positive Glucotropaeolin—positive	Döring and Ulber (2020); Bartlet et al. (1994 and 1996)
	Crucifer flea beetle *(Phyllotetra cruciferae)*	Increased: sinalbin, gluconapin Decreased: progoitrin	Sinalbin—negative Glucoheirolin—neutral Glucocapparin—positive Glucoiberin—positive Glucotropaeolin—positive Sinigrin—positive	Bodnaryk (1991); Hicks (1974); Soroka and Grenkow (2013)
Lepidoptera	Diamondback moth (*Plutella xylostella*)	Increased: none Decreased: total GSL, glucobarbarin, gluconasturtiin	Allyl isothiocyanate—negative Sinigrin—neutral Glucobarbarin—positive Gluconasturtiin—positive	Badenes-Pérez et al. (2020 and 2014); Hariprasad and van Emden (2010); Li et al. (2000)
	Bertha armyworm (*Mamestra configurata*)	Increased: isothiocyanate-releasing GSL, sinalbin, sinigrin Decreased: none	Allyl isothiocyanate—negative Sinalbin—negative Sinigrin—negative Indole-3-carbinol—neutral	McCloskey and Isman (1993); Bodnaryk (1991)
	Cabbage moth (*M. brassicae*)	Increased: total GSL, aliphatic GS, total indolic GLS, gluconapin, sinigrin, glucoiberin, glucobrassicin Decreased: sinigrin, glucoiberin	Glucobarbarin—negative Gluconasturtin—negative	Gols et al. (2008b); Ahuja et al. (2015); Santolamazza-Carbone et al. (2016); Badenes-Pérez and Cartea (2021); Müller et al. (2018)
	Turnip sawfly (*Athalia rosae*)	Increased: indole GSL Decreased: aliphatic GSL	Glucosinalbin– neutral	Abdalsamee and Müller (2012); Müller (2009)
	Small and large white butterfly (*Pieris brassicae* and *P. rapa*)	Increased: glucoiberin Decreased: indole GSL, neoglucobrassicin, glucoiberin, glucobrassicin, sinigrin	Propenyl isothiocyanates—positive	Mithen (1992; and references therein); Gols et al. (2008b); Santolamazza-Carbone et al. (2016)
Diptera	Turnip root fly (*Delia floralis*)	Increased: none to total GLS Decreased: none to total GLS	Progoitin—neutral Glucoerucin—neutral Glucoiberin—neutral Neoglucobrassicin—neutral Glucobrassicin—positive Glucobrassicanapin—positive Gluconapin—positive Gluconastrurtiin—positive & negative Glucotropaeolin—positive & negative Sinalbin—positive Sinigrin—positive	Birch et al. (1992); Hopkins et al. (1997); Simmonds et al. (1994), Gouinguené and Städler (2006)
	Cabbage root fly (*Delia radicum*)	Increased:— Decreased: benzyl and indolyl glucosinolates, glucobrassicin	Glucobrassicanapin—neutral Gluconapin—neutral Gluconasturtiin–neutal Glucotropaeolin—neutral Progoitrin—neutral Sinigrin—neutral Glucobrassicin—positive	Roessingh et al. (1992); Gouinguené and Städler (2006)
Sternorrhyncha	Cabbage whitefly (*Aleyrodes proletella*)	Increased: none to total and single GLS, sinigrin Decreased: none to total and single GLS		Hondelmann et al. (2020); Newton et al. (2010)
	Green peach aphid (*Myzus persicae*)	Increased: indole GSLs, total GSL, gluconapin Decreased: none, progoitrin, sinalbin, glucobrassicin	Di-indolyl-methylcysteines—negative Glucobrassicin—negative Indolyl-methylcysteine—negative	Kim et al. (2008); Kim and Jander (2007); Mezgebe and Azerefegne (2021); Cole (1997)
	Cabbage aphid (*Brevicoryne brassicae*)	Increased: total GSL, indole GSLs, gluconapin, glucobrassicin Decreased: progoitrin, sinalbin	Sinigrin—positive & negative	Kazana et al. (2007); Newton et al. (2010); Mezgebe and Azerefegne (2021); Cole (1997)
	Mustard aphid (*Lipaphis erysimi*)	Increased: Hydroxy-glucobrassicin, total GLS Decreased: gluconapin		Dilawari et al. (2003); Kumar et al. (2017)

Besides these feeding related glucosinolates, Brassicaceae emit volatile glucosinolate breakdown products such as isothiocyanates, thiocyanates, nitriles, and epithionitriles (Halkier and Gershenzon 2006). The emission of constitutive oilseed rape VOCs (volatile organic compounds), i.e. volatiles emitted by the undamaged plant, could be manipulated by breeding to achieve a lower emission of pest-attracting VOCs. For instance, different Brassicaceae species vary in their attractiveness to the pollen beetle for oviposition. These differences can be attributed mostly to scent compounds from the flower buds, which are preferred by the winter generation of the beetle (Ruther and Thiemann 1997; Jönsson et al. 2007). To reduce the olfactory attraction of pollen beetle to oilseed rape, the biosynthesis of 2-phenylethyl, 3-butenyl and 4-pentenyl isothiocyanate, phenylacetaldehyde, and indole could be reduced by breeding for a lower content of alkenyl glucosinolates (reviewed in Mauchline et al. 2018; Cook et al. 2007a; see above). Isothiocyanates (2-phenylethyl isothiocyanate), together with nitriles (phenylacetonitrile, 4-pentenenitrile and 5-hexenenitrile) and other volatiles play a role in host plant recognition and olfactory attraction of the seed weevil *C. assimilis* (Bartlet et al. 1993; Tansey et al. 2010a). On the other hand, increasing the constitutive emission of attractive volatiles could render certain Brassicaceae plants ideal trap crops when planted on oilseed rape field borders. For instance, *B. rapa* is particularly attractive to *B. aeneus* due to its development cycle and scent composition including compounds such as phenylacetaldehyde, indole and (E,E)-*α*-farnesene (Cook et al. 2007b).

The major bottleneck for breeding for modified GSL content in non-seed organs of oilseed rape to enhance insect pest resistance is currently the lack of simple and cost-efficient high-throughput analytical methods for individual GSL. For seed GSL profiles, NIRS analysis has been applied broadly. For non-seed organs, a widely distributed method is based on reversed phase high-performance liquid chromatography (RP-HPLC) which is time- and labour-consuming and requires special equipment (Ishida et al. 2014). NIRS-based methods for quantifying GSL profiles and individual GSL in stems and leaves have been evaluated in Brassicaceae species and oilseed rape, but not broadly applied in breeding (Font et al. 2005; Toledo-Martín et al. 2017) and should be further developed for oilseed rape. Beckman et al. (2007) reported a preliminary NIRS calibration for leaf GSL in a reduced set of *B. napus* genotypes with good prediction accuracies at least for the total GSL contents and showed the potential of this method as an HTP selection tool for *B. napus* genotypes with altered leaf GSL profiles. Because these methods dealing with vegetative tissues might require freeze drying or handling of samples under cool conditions of samples, it will allow less throughput compared to NIRS analysis of seeds, but will allow a substantially higher throughput than sample preparation and analysis by HPLC of stem and leave or other non-seed organs of oilseed rape.

Many genes involved in GSL biosynthesis and their genetic controls are well described in the model plant *A. thaliana*, in *B. napus* and other Brassicaceae species (Kittipol et al. 2019; Mitreiter and Gigolashvili 2021). Mutations within GSL biosynthesis genes which disrupt expression can be efficiently identified by TILLING-by-Sequencing approaches (see “Transgenic, genome-engineering and mutation breeding approaches” section). The genomic and genetic knowledge together with the genetic resources and the perspectives for developing simple HTP phenotyping methods makes modified glucosinolate content as a proxy for insect resistance an excellent breeding target with a short-term perspective (Table 2, Fig. 1).

### Breeding for modified profiles of other secondary plant metabolites

*Brassica* plants contain, in addition to glucosinolates, many other groups of secondary metabolites which are sometimes lineage specific. Secondary metabolites can have multifunctional roles and are often associated with adaptive evolution to changing environments including regulation of growth, development and plant defence (Erb and Kliebenstein 2020). Major groups of secondary metabolites in the Brassicaceae involved in interaction with insects include terpenoids, phytosterols, flavonoids, phenolics, cyanogenic compounds and alkaloids (Hegedus and Erlandson 2012). A meta-analysis of plant–insect interactions including diverse plant taxa showed that higher foliar content of flavones and saponines are mainly involved in anti-herbivore defence at the constitutive level, whereas upregulation of anthocyanins, flavonoids, quinones, alkaloids and other compounds are involved in induced defence (Sardans et al. 2021). One of the largest groups of these metabolites are phenolics originating from the shikimate–phenylpropanoids–flavonoids pathways (Lattanzio et al. 2006). There is a multitude of reports on the role of polyphenols and secondary metabolites in plant–insect interactions and plant defences for diverse plant species (e.g. Singh et al. 2021). However, studies that have validated the anti-herbivory effects of secondary metabolites in crops under field situations are very limited (Kortbeek et al. 2019). A literature review on secondary metabolites for which anti-insect activity has most often been reported across all plant species revealed isoprene-derived terpenoids followed by alkaloids and phenolic compounds at the first 3 positions (Boulogne et al. 2012). Most of the studies on Brassicaceae species are addressing the effects of glucosinolates (e.g. Kliebenstein 2014; Kumar 2017). Detailed functional studies on the anti-herbivory effects of other secondary metabolites in oilseed and vegetable *Brassica* species are scarce (e.g. Ahuja et al. 2010). Terpenoids including triterpene saponins play a role in specialist insect resistance in the Brassicaceae genera *Lunaria* (honesty), *Thlaspi* (pennycress) and *Barbarea* (winter cress) for resistance against the striped flea beetle (*Pyllotetra striolata*), the diamondback moth*,* the cabbage butterfly (*P. rapae*) (reviewed in Hussain et al. 2019) and pollen beetles (Austel et al. 2021). Tetracyclic triterpene steroids from *Iberis* species (candytuft) are involved in resistance against several species of flea beetles (Nielsen 1978) as well. Because these Brassicaceae species cannot be crossed with *B. napus*, generation of resistance in oilseed rape would require a gene transfer approach. Phytosterols including brassicasterol are found in *B. napus* and have been shown to adversely affect the insect species Bertha armyworm (*Mamestra configurata*) and crucifer flea beetle (*Phyllotetra cruciferae*), but not the green peach aphid (Hegedus and Erlandson 2012). Diverse types of flavonoids are bioactive against specialist insect herbivores in *Brassica* species and *B. napus*. However, some show adverse effects on some insect species while being stimulatory to other insect species, e.g. quercetin and/or kaempferol glycosides and their derivates deterred the Bertha armyworm and the cabbage seedpod weevil, but stimulated the diamondback moth and the horseradish flea beetle (*P. armoraciae*) (Hegedus and Erlandson 2012; Lee et al. 2014). *B. napus* leaves contain only traces of quercetin glycosides, but large amounts of kaempferol glycosides (Gruber et al. 2018), which would enable targeted breeding for increased quercetin glycoside levels. The phenylpropanoid ester chlorogenic acid confers broad-spectrum resistance against many pathogens and insect pests in many plant species including *B. napus* (Lattanzio et al. 2006; Obermeier et al. 2013; Kundu and Vadassery 2019; Singh et al. 2021). Sinapic acid and its derivates are highly abundant in different tissues of *Brassica* species, especially in seeds. These compounds are involved in multiple biological processes including adaptation to stress and protection against UV radiation (Nguyen et al. 2021). Sinapic acid has antimicrobial activities. It also has been described to reduce spruce budworm (*Choristoneura fumiferana*) oviposition (Grant and Langevin 2002) and cabbage root fly (*Delia radicum*) oviposition on cauliflower (*B. oleracea*) (Jones et al. 1988). Sinapic acid is a precursor of sinapate esters. Sinapate esters have been shown to be involved in disease resistance against the soil-borne pathogen *Verticilllum longisporum* in Arabidopsis (König et al. 2014). The sinapate ester sinapoyl malate was shown to be induced in *B. rapa* by herbivory of both larvae of the generalist beet armyworm (*S. exigua*) and the specialist diamondback moth in leaves (Widarto et al. 2006). Tannins are polyphenolic compounds showing antifungal activity against many filamentous fungi and are at the same time deleterious to many phytophagous insects (Lattanzio et al. 2006). However, no reports for the anti-herbivory activity of tannins or condensed tannins (proanthocyanidins) in *Brassica* species and *B. napus* exist. The alkaloid camalexin is involved in resistance of Arabidopsis against fungal pathogens, against the green peach aphid and against the cabbage aphid (Kettles et al. 2013; Kuśnierczyk et al. 2008). Only very few plant species of the Brassicaceae family are known to produce cyanogenic compounds. However, the cyanogenic compound alliarinoside found in the *Brassica* species *Alliaria petiolata* (garlic mustard) has been described to strongly inhibit the feeding of the Mustard White butterfly (*Pieris napi oleracea*) (Renwick et al. 2001). Resistance to the flea beetle *Pyllotetra nemorum* has been introduced to Arabidopsis by transferring the entire pathway for synthesis of the cyanogenic glucoside dhurrin from *Sorghum bicolor* (Tattersall et al. 2001). Glucosinolate breakdown products are implicated in callose accumulation, a polysaccharide produced to enhance cell walls after stress or damage induction (Erb and Kliebenstein 2020). Callose accumulation is involved in resistance to phloem-sucking aphids and has been well studied in Arabidopsis (Kuśnierczyk et al. 2008; Shoala et al. 2018). In *B. rapa* and *B. nigra* callose accumulation also has been induced by the cabbage butterfly (Caarls et al. 2021; preprint).

Oilseed rape and other *Brassicaceae* emit various mono- and sesquiterpenoids, green leaf volatiles and benzenoids (Himanen et al. 2017; Dudareva et al. 2013). While oilseed rape is limited in (volatile) chemical diversity due to a long breeding history with specific bottlenecks (Mason and Snowdon 2016), other *Brassicaceae* species offer a higher diversity of volatile compounds. Breeding could aim for increasing the emission of specific VOCs that act as repellents for non-adapted herbivores. Plant species that are phylogenetically distant from *B. napus*, and repel *Brassica* specialists, might be sources for breeding (Stratton et al. 2019). It is known that some Brassicaceae genera like *Raphanus* and *Eruca* are less attractive and cause high larval mortality in the pollen beetle (Veromann et al. 2014). However, for most of these *Brassicaceae* species interspecific crossing success is low. Thus, it has been suggested that by using GMO approaches repellent non-host volatiles from non-Brassicaceae species such as *Lavandula* spp. or *Mentha* spp. could be introduced, subsequently masking host plant volatiles of *B. napus* to reduce its attractiveness to herbivores (reviewed in Mauchline et al. 2018; Li et al. 2019; Guo et al. 2020).

Companion plants can alter the VOC environment encountered by pests, and cultural control can involve trap crops and intercrops, with VOC emissions, potentially optimised by breeding. Pollen beetles, for instance, are attracted by visual and olfactory cues of *B. rapa*, which was successfully used as trap crop surrounding plots of *B. napus* (Cook et al. 2007a). Optimising VOC blends in oilseed rape can be achieved through classical or precision plant breeding or through genetic modification of VOC biosynthetic pathways, including new breeding techniques such as CRISPR/Cas (Chen et al. 2019).

Phenotyping for most secondary metabolites is based on analytical methods like HPLC and GC–MS, which require specialised laboratory equipment and expertise and are comparatively slow and expensive. In addition, some secondary metabolites are degraded after plant material is sampled and require freeze-drying of the material before analytical processing. This represents a severe bottleneck for high-throughput in breeding. Due to the multifunctional roles of most secondary metabolites, breeding for a modified profile of secondary metabolites with enhanced insect resistance has also to consider the potentially negative aspects for development, quality and yield of oilseed rape (e.g. Ahuja et al. 2010). There is a knowledge gap for the multifunctional roles and pleiotropic effects of most secondary metabolites, which requires more research before integration into commercial breeding is reasonable. Currently, the most promising secondary metabolites for integration into breeding programs towards insect resistance seem phenylpropanoid esters, e.g. chlorogenic acid. For breeding sweet potato for insect resistance, it has been demonstrated that a rapid and reliable analytical technique for chlorogenic acid based on thin layer chromatography can be adopted for screening large accessions of breeding material (Lebot et al. 2021). The current lack of advanced knowledge on the diverse multiple functions and variation in secondary metabolites in oilseed rape together with the lack of high-throughput phenotyping methods make breeding for modified secondary metabolite profiles for insect resistance a breeding target with a medium- to long-term perspective (Table 2, Fig. 1).

### Breeding for modification of plant surfaces and enhancement of physical barriers

The leaf or stem surface is the first physical barrier an aboveground herbivore is facing during close range host plant orientation. The waxiness and thus the adhesiveness of the plant surface as well as the tissue toughness and hairiness are three important physical properties. Glossy (waxless) phenotypes of *Brassica* ssp. can lead to increased resistance in various insect species (e.g. for *L. erysimi* and *B. brassicae*) as well as to increased susceptibility (e.g. greater feeding damage by the crucifer flea beetle *P. crucifera)* (Stoner 1992; Eigenbrode and Espelie 1995; Hegedus and Erlandson 2012; Lammerts van Bueren et al. 2021). Leaf toughness has a negative effect on feeding damage by chewing herbivores (Choong 1996; Malishev and Sanson 2015; Caldwell et al. 2016), whereas sap sucking or mining insects are mostly not affected (Caldwell et al. 2016). This has been demonstrated for several plant taxa, but no studies have been conducted for the Brassicaceae (Caldwell et al. 2016; Hao et al. 2020). Due to contrary effects on herbivores, breeding for modified epicuticular wax composition or leaf toughness might not be a suitable breeding target to create oilseed rape with resistance against multiple insect pests, although a reduced wax bloom has positive effects on predatory insects (see “Breeding for manipulation of natural enemies of insect pests” section).

Non-glandular trichomes have been shown to be efficient physical barriers against herbivorous pests for a number of plant species within the Brassicaceae family (Müller 2006; van Poecke 2007), e.g. against *P. xylostella* (Løe et al. 2007) and flea beetles (Brown et al. 1997). Contrasting reports exist whether the degree of specialisation of herbivores to *A. thaliana* is associated with the involvement of trichome density in plant defence (Handley et al. 2005; Pfalz et al. 2007). A number of genes involved in trichome formation, trichome density and its modulation by environmental factors have been identified and well characterised in *A. thaliana* (Hülskamp 2004; Hauser 2014; Vadde et al. 2019). Trichome density can be increased after induction by herbivore attack in Brassicaceae species (Traw and Dawson 2002; Travers-Martin and Müller 2008; Mathur et al. 2011, 2013). Genes involved in trichome formation have also been characterised in *R. sativus* (Li et al. 2013), *B. villosa* (Nayidu et al. 2014), *B. rapa* and *B. napus* (Tian et al. 2018). Molecular markers useful for marker-assisted selection in breeding have been identified in *B. oleraceae* (Mei et al. 2017) and in *B. napus* (Xuan et al. 2019). Genetic transfer of one of the *A. thaliana* genes involved in hairiness into *B. napus* has resulted in ultra-hairy lines showing strong feeding deterrence to crucifer flea beetles and modest resistance to larvae of the diamondback moth (Soroka et al. 2011; Hegedus and Erlandson 2012; Alahakoon et al. 2016a, 2016b; Gruber et al. 2018). However, growth in these genetically modified *B. napus* plants was negatively affected. In the past, most studies have focused mainly on trichome formation on leaf surfaces. Future studies are required that tackle trichome formation on shoots to efficiently target stem flea beetle and stem weevil resistance, on flower buds to target pollen beetle resistance or on seed pods to target bug and weevil resistance. Furthermore, the role of trichomes is not limited to providing physical barriers to insects; they can also mediate chemical defence as well. From *A. thaliana* it is known that non-glandular trichomes accumulate phenylpropanoids and glucosinolates (Sinlapadech et al. 2007; Jakoby et al. 2008).

A high-throughput phenotyping method for automated assessment of trichome density on leaves has been developed for grapevine (Divilov et al. 2017) which could be adapted to oilseed rape. The extensive molecular knowledge of trichome development in *A. thaliana* and the availability of molecular markers from *Brassica* species and HTP technologies makes hairiness of leaves and other plant organs a promising trait for breeding oilseed rape with resistance to flea beetles and other insect pests with a good short-term perspective (Table 2, Fig. 1).

Other herbivore-induced physical defence mechanisms which might be exploitable to create insect-resistant oilseed rape cultivars in the future are insect-egg killing traits, including the expression of hypersensitive response (HR)-like necrosis by the host plant on the leave beneath deposited Lepidopteran and Coleopteran eggs. Because egg-killing traits kill an immobile stage of the pest before feeding damage occurs, breeding for this trait might provide a highly effective plant protection strategy in cases where larvae cause significant feeding damage (Fatouros et al. 2016). Due to the expression of HR-like necrosis, eggs desiccate and/or drop off from the plant. This type of defence has been described for several plant taxa including Brassicaceae. An egg-killing effects has been described in *B. nigra* against the eggs of the cabbage white butterflies, *Pieris rapae* and *Pieris brassicae* displaying variation in different wild black mustard populations (Fatouros et al. 2014; Griese et al. 2017, 2021). Against the cabbage moth (*Mamestra brassicae*) and the diamondback moth, the insect-killing traits were found to be less effective in *B. nigra* (Griese et al. 2021). In *B. napus,* HR-like necrosis with a less severe phenotype has been observed in 9 out of 10 tested genotypes against *P. brassicae* eggs considered as intermediate–strong HR responses (Griese et al. 2021; Afentoulis et al. 2021).

### Breeding for manipulation of natural enemies of insect pests

Predators and parasitoids constitute an important dimension of natural pest control. Conceptually, tri- or multitrophic interactions across crop plants, pest insects and their natural antagonists are mediated by a multitude of plant and insect traits which may provide breeding targets to improve natural pest control and may represent promising strategies in a pesticide free agriculture (Agrawal 2000). Direct plant defences against insect pests and plant volatiles mediating interactions between plants and pests have been discussed elsewhere in the manuscript (see above). In addition, there are several rather basic plant traits such as physical and chemical plant characteristics affecting natural antagonists. It has been shown that very basic plant characteristics such as plant size can affect herbivores and interactions of herbivores, pollinators and natural enemies (see Schlinkert et al. 2015, 2016 for examples from the Brassicaceae), and it is well known that size and plant architecture influence the interactions between herbivores and their natural antagonists (see Frazer and McGregor 1994; and additional references in White and Eigenbrode 2000). On a smaller scale, especially plant surface traits such as cuticular waxes and trichomes appear to be critical determinants of the efficiency of predators and parasitoids of herbivores (see “Breeding for modification of plant surfaces and enhancement of physical barriers” section). An example from the Brassicaceae includes the interaction of 4 ladybird species with the cabbage aphid across 3 species of *Brassica* and *Hirschfeldia incana* (shortpod mustard). While the predation rates across ladybird species were not different, there was a difference across plant species. Among other differences, especially the frequency of falling off mediated by the plant surface differed strongly across plant species (Grevstad and Klepetka 1992). It has been demonstrated mechanistically, that the attachment force of predatory lacewing larvae depends on the waxiness of *B. oleracea* leaves. On cabbage leaves with a glossy phenotype (‘glossy’ mutations, leading to a reduced wax bloom) adhesive force was 20 to 200-fold greater compared to phenotypes with a normal wax bloom. This pattern was reflected by the predation efficiency on larvae of the Brassicaceae pest *P. xylostella* which was substantially higher on glossy leaves (Eigenbrode et al. 1995). The effect of trichomes on predatory insects have been reviewed by Riddick and Simmons (2014) over a wide range of agricultural plants, but not in Brassicacea. They concluded that trichomes have more harmful than beneficial effects on predatory insects, but most harmful effects are sublethal.

Attracting natural enemies (parasitoids, predators) by volatiles is another plant protection strategy in oilseed rape, which could be exploited by breeders in the future. A dozen parasitoid species of the most common oilseed rape pests are sufficiently widespread and abundant across Europe to be of potential economic importance for conservation biological control (Ulber et al. 2010). Attracting natural enemies involves the emission of herbivore-induced plant volatiles (HIPVs). In addition to the emission of constitutive volatiles from undamaged plants, oilseed rape and other Brassicaceae species typically respond to the oviposition or feeding activity of insect herbivores by releasing a range of volatiles into the environment (Himanen et al. 2017). Such HIPVs are emitted in greater quantities compared to undamaged plants, and the HIPV mixture provides a chemical signal that predatory and parasitic insects can use to locate an individual plant on which their prey or host is located (Heil 2014; Hilker and Meiners 2006; Turlings and Erb 2018). In *B. napus,* leaf feeding by diamondback moth caterpillars leads to higher emission rates of various monoterpenes such as *α*-thujene, sabinene, limonene, the homoterpene dimethylnonatriene, or the sesquiterpenes *β*-elemene and (*E,E*)-*α*-farnesene (Himanen et al. 2009; Ibrahim et al. 2008). The induced HIPV mixture is very attractive to the solitary wasp *Cotesia vestalis* (Himanen et al. 2009). Similar results have been reported with pollen beetle-infested *B. napus* and several of its parasitoids. However, in this case HIPVs were collected from plants at the bud stage, resulting in a much richer mixture containing flower-specific volatiles in addition to HIPVs released from vegetative tissues (Jönsson and Anderson 2008; Jönsson et al. 2005). Such specificity of HIPV mixtures indeed plays an important role in HIPV-mediated interactions, as natural enemies rely on the correct odor gestalt to find their prey or host (Bruce and Pickett 2011). This issue of specificity is particularly relevant for specialised parasitoids, whereas generalists tend to learn existing odors as conditioned stimuli when they encounter their victims. Understanding the information content of specific HIPV mixtures for carnivorous insects in a tritrophic environment is therefore a prerequisite before breeding for improved HIPV emission can commence. The quantity and composition (quality) of the HIPVs emitted by a given plant depend on several factors, including the herbivore species, its feeding mode (Rowen and Kaplan 2016; Turlings and Erb 2018), the infested plant part, the age of the plant, or the simultaneous presence of herbivores and microbes. Abiotic stresses such as drought, nutrient availability, soil salinity, or ozone concentration have been found to alter the amount of all or some HIPVs. However, such changes do not always reduce the attractiveness of the altered mixture to natural enemies (Becker et al. 2015; Wäschke et al. 2013). Several studies have demonstrated strong intraspecific differences in HIPV mixtures that result in differential attraction to natural enemies (Aartsma et al. 2019; Degen et al. 2004; Kappers et al. 2011). Domestication and breeding efforts may inadvertently have resulted in modern plant genotypes that emit HIPV mixtures with relatively low attraction potential (Rasmann et al. 2005; Tamiru et al. 2011). Glucosinolate-derived HIPVs play a role in the attraction of the third trophic level in the Brassicaceae (Hopkins et al. 2009). Wild and cultivated *B. oleracea* showed significant differences in HIPV mixtures and attraction to the cabbage whitefly parasitoid *Cotesia rubecola*. It was suggested that isothiocyanates, volatile degradation products of glucosinolates emitted only by wild *B. oleracea*, might be the key compounds (Gols et al. 2011). A meta-analysis of HIPVs emitted by Brassicaceae and Solanaceae species revealed that domesticated species in general release higher amounts of green leaf volatiles and terpenes than wild species, but their HIPV blends are less divers (Rowen and Kaplan 2016). The latter point seems to be crucial for pest control, since increased emission per se does not necessarily affect natural enemies. It is rather the presence or absence of specific compounds and the ratio of volatiles that are important for attraction (D’Alessandro et al. 2006; Bruce and Pickett 2011; Beyaert et al. 2010). When aiming for improving the attraction of oilseed rape to predators or parasitoids one option is the improvement in the production and emission of HIPVs by breeding, maybe even by changing it from an inducible to a constitutive mode (van Lenteren 1986). For example, modified cis-jasmone concentrations are activating defence and volatile emission in a range of cultivars of *B. napus*, *B. rapa* and *B. oleracea* and are making these plants less attractive to and less suitable for the aphid *M. persicae*, but more attractive to the parasitoid *Diaeretiella rapae* (Ali et al. 2021).

In general, breeding for specific HPVs or constitutive volatile compounds needs to take environmental factors into account that may affect the emission and stability of the volatile signals. Simultaneously, these factors may affect the responding insects as well. Knowledge on these interactions needs to be collected and provided by biologists as the consequences of manipulating specific volatiles has to be evaluated in a broader ecological context. Changing volatile emission might not only repel herbivores or attract natural enemies more efficiently. As an unwanted side effect, it could also promote the success of plant pathogens. For example, Arabidopsis mutants with increased monoterpene emission were more susceptible to *Verticillium longisporum* than wild type plants (Roos et al. 2015). Changing volatile emission by breeding can also lead to other unwanted side effects and might negatively affect successful habitat or host location of predators, parasitoids or pollinators. A conventionally bred-resistant cultivar of Brussels sprout supported lower parasitism rates of the cabbage aphid because the plant produced less of specific glucosinolate precursors of volatile cues, which reduced its attractiveness to females of the aphid parasitoid *Diaeretiella rapae* (van Emden 1995). Metabolic engineering of plant volatiles can furthermore lead to less attractive flowers for pollinators (Dudareva et al. 2013). It can also cause elevated human or animal consumer irritations or allergies due to the enhanced production of allergenic compounds (McEwan and Macfarlane Smith 1998). Another option is the modification of plant VOC emission and natural enemy attraction via exogenous application of defence elicitors or plant strengtheners (Rostás and Turlings 2008; Sobhy et al. 2014). The responsiveness of oilseed rape to these elicitors could also be an aim of breeders.

Besides indirect defences via natural enemy attraction, secondary compounds involved in plant tolerance or resistance can indirectly affect the 3rd trophic level via the sequestration of plant compounds as a defence by insect pests (Hopkins et al. 2009; Opitz and Müller 2009; Petschenka and Agrawal 2016). Remarkably, several insects specialised on Brassicaceae are known to sequester glucosinolates including important pest species such as the cabbage aphid and the mustard aphid (Kazana et al. 2007), the turnip sawfly (*A. rosae*) (Müller and Wittstock 2005), and flea beetles (*P. chrysocephala*, *P. armoraciae, P. striolata*) (Beran et al. 2014, 2018; Sporer et al. 2020). While glucosinolates in plants are known to confer tolerance against some insects (Hopkins et al. 2009; Jeschke et al. 2017), they have been shown to impair predators (Kos et al. 2012; Sun et al. 2019) and can even affect the fourth trophic level (hyperparasitoids) (Harvey et al. 2003). Consequently, breeding to reinforce glucosinolates in oilseed rape as a defence against herbivores may negatively affect biocontrol of specialised insects (Gols and Harvey 2009). In line with this, the survival of predatory ladybird larvae (*Adalia bipunctata*) depends on the *Brassica* host plant species of its specialist aphid prey *B. brassicae* and may be mediated by the plants’ glucosinolate content. In contrast, for the generalist aphid pest *M. persicae* no such differences across *B. napus*, *B. nigra* and *S. alba* plant species were observed (Francis et al. 2001). Plant-mediated effects on predators are not only observed across plant species but also occur within *Brassica* species. Across four cultivars of white cabbage (*B. oleracea*), Kos et al. (2011) found differences in the performance of the hoverfly *Episyrphus balteatus* and the lacewing *Chrysoperla carnea* preying upon the aphid *B. brassicae*. Moreover, differences in the performance of predators matched differences in the glucosinolate profiles sequestered by the aphids. Across three populations of wild *B. oleracea*, the performance of the endoparasitoids *Cotesia rubecula* and *Microplitis mediator* reflected the performance of their hosts, the cabbage butterfly and the cabbage moth which was affected by plant quality (Gols et al. 2008b). A screening across 10 oilseed rape/canola cultivars revealed > 35-fold differences in fecundity of the cabbage aphid between the most susceptible and the most resistant cultivar. A comparison between the most susceptible and one of the most resistant oilseed rape cultivars revealed that performance of the aphid decreased by 93% while the performance of its parasitoid *Diaeretiella rapae* only decreased by 20% (Karami et al. 2018).

Currently, methods for high-throughput screening for use in breeding to characterise phenotypes affecting tri- or multitrophic interactions are not available. The efficiency of parasitisation and predation could be assessed in the future by video-based solutions. Experiments involving video technology for observing several replicates of predation experiments simultaneously were already described 20 years ago (Meyhöfer 2001) and can be used to observe parasitoid behaviour under field conditions (de Bruijn et al. 2021) or for high throughput recording of insect behaviour (de Bruijn et al. 2018). Especially experiments with invertebrate predators such as lacewing larvae can be carried out in small Petri dishes (Pokharel et al. 2020) and probably even multiwell plates and thus would allow the conversion into a high throughput screening methods. Other traits influencing the efficiency of natural antagonists such as the attachment force of predators on leaf surfaces could be quantified via simple centrifugal devices as described by Eigenbrode et al. (1999). Although we have good knowledge about multiple traits mediating tritrophic interactions in the Brassicaceae, quantification of variation across genotypes will require the development of novel technologies for high throughput screening, and therefore, most of the traits outlined above can be integrated into breeding only under a long-term perspective (Table 2, Fig. 1).

### Breeding for an improved microbiome

The integration of plant microbiomes for improved crop protection has been advocated frequently within the last years (e.g. Sessitsch and Mitter 2015; Trivedi et al. 2021). A number of approaches for manipulating the microbiome which are useful for insect pest management have been identified and future opportunities for the discovery of new biopesticides have been described, including plant-derived protectants and semiochemicals (Qadri et al. 2020). Direct or indirect selection and breeding for improved plant–microbe interactions to enhance biotic stress tolerance has been suggested as a new concept to manipulate the microbiome of crop plants (e.g. Gopal and Gupta 2016; Clouse and Wagner 2021). Microbiome-targeted approaches for crop improvement have been classified as first-generation approaches defined as applying single microorganisms to improve plant growth or control pathogens and pests. These approaches have been applied for more than 100 years. In contrast, second-generation future approaches have been defined as engineering of whole microbial communities and breeding of crop plants for enhanced interaction with beneficial microorganisms (French et al. 2021). Microbiome research has focused in the past mainly on plant-associated below-ground microbiome composition and less on above-ground microbiome composition with regard to plant interaction with pathogens and insect pests. For the involvement in the interaction of plants and insects, most recent studies have been focusing on the soil and rhizosphere microbiome and its indirect manipulation (e.g. by crop rotation and breeding) has been suggested as a target for suppression of aboveground insect pests (Pineda et al. 2017).

Currently, quantitative data generated from next-generation sequencing approaches are the most suitable high-throughput proxy traits for microbiome phenotyping. Mostly amplicon next-generation sequencing of variable regions of conserved marker genes like bacterial 16S rRNA genes or fungal Internal Transcribed Spacer regions of rRNA genes are used (Beilsmith et al. 2019). However, the microbial community-wide sequence data generation within large plant populations for genetic mapping of plant loci involved in controlling the abundance of specific microbial taxa under different environmental conditions is still in its infancy and HTP approaches useful for commercial breeding are not available yet. Until now, 16S rRNA next-generation sequencing data from plant populations has only been used in Arabidopsis and sorghum to identify genetic loci controlling whole phyllosphere and rhizosphere bacterial and fungal communities by genome-wide association analysis (Horton et al. 2014; Deng et al. 2021). To substantially extend these kinds of studies in the future in oilseed rape and other crops, it is necessary to identify and further characterise putative target loci for breeding towards an improved microbiome.

The oilseed rape rhizosphere and root microbiomes are different from the microbiomes of other crops (Lay et al. 2018; Morales Moreira et al. 2021). Its composition in rhizosphere, roots, leaves and seeds depends on a combination of factors including environmental conditions, agronomic treatments (e.g. fertilisation and seeding density), plant developmental stage and is also cultivar-dependent (e.g. Rybakova et al. 2017; Taye et al. 2020; Morales Moreira et al. 2021). Taye et al. (2020) reported that across different oilseed rape growth stages in the field 16–37% of the variation between 16 diverse *B. napus* genotypes was either directly or indirectly due to genetics which would allow to manipulate its composition through targeted breeding. Studies on the composition and genotype-specific association of the oilseed rape microbiome with resistance against pathogens and insects are rare. It has been shown that root endophytic bacterial communities of oilseed rape or its soil microbiome are associated with genotype-specific resistance against the soil-borne fungal pathogen *Verticillium longisporum* (Glaeser et al. 2019) and *Plasmodiophora brassicae* (Daval et al. 2020). The soil-borne endophyte *A. alternatum* is producing phytosterols in *B. oleracea* which has been shown to inhibit the growth of *P. brassicae* and the diamondback moth (Jäschke et al. 2010; Raps and Vidal 1998). The influence of the soil microbiome composition on development of the cabbage root fly in roots and above-ground parts of oilseed rape was only marginal (Lachaise et al. 2017). In *B. oleracea*, in contrast, resistance to the cabbage root fly was significantly reduced by rhizobacterium *Pseudomonas simiae* added to the soil (Friman et al. 2021). For *Boechera stricta* (Drummond’s rockcress), it has been shown that the disruption of the rhizosphere microbiome composition increases the susceptibility against the green peach aphid and the crucifer flea beetle (Hubbard et al. 2019). Aside from a few studies on Brassicacea discussed above, no other research exists   describing plant–microbiome–insect interaction in relation to oilseed rape genotypes.

For a number of reason the most promising actual target for microbiome manipulation through breeding for insect resistance seems to be the seed microbiome. First of all, it has been shown for *B. napus* that a large component of the seed microbiome is cultivar dependent (Morales Moreira et al. 2021). Second, *B. napus* cultivars harbouring higher indigenous seed microbiome diversity were characterised as having a higher colonisation resistance against beneficial and pathogenic microorganisms (Rybakova et al. 2017). Third, *B. napus* seeds have been shown to be involved in the vertical submission of endophytic microbiota to seedlings and the next generation (Rochefort al. 2021). Finally, seeds are easy to sample and analyse in high throughput assays adaptable to commercial breeding schemes. The plant genotype together with the cropping environment has an impact on the seed microbiome composition in *B. napus* (Rochefort et al. 2019). However, the seed microbiome is a lot less diverse and abundant compared to the rhizosphere and root microbiome. Transmission of microbiota to the roots and shoots of the seedling is driven mainly by the soil microbiome, but to a much lower extent by the seed microbiome due to a soil microbial mass effect. However, *B. napus* is selecting rare seed-borne microbiota in the seedlings which suggests that these rare taxa increase the fitness of the plants (Rochefort et al. 2021). For barley, a plant genotype-dependent endophytic seed microbiome was found with most of the isolated endophytes showing diverse plant beneficial characteristics in vitro (Bziuk et al. 2021). The feasibility of this concept and its large economic success has been shown in cool season grass (*Lolium perenne*) breeding in New Zealand, Australia and the USA. Cool season grass cultivars have been successfully bred and commercialised whose seeds are inoculated with compatible strains of the fungal endophyte Epichloë. These endophyte strains are producing selected alkaloids (pyrrolizidines) in the plants with anti-herbivore effects without the toxic effects on animals and other vertebrates (Caradus and Johnson 2020; Lee et al. 2021). In addition, for many Brassicaceae species the association with beneficial endophytic microorganisms has been shown (Card et al. 2015). Detailed studies and understanding of seed-transmitted microbiota and their genotype-specific influence on plant performance might enable a targeted breeding strategy towards seed microbiome mediated insect resistance in oilseed rape.

Breeding for an improved microbiome firstly requires understanding of host genetic control of the microbiome in different plant organs, its variability and its interaction with insect performance in different genotypes under field conditions. The current lack of knowledge on the genetics, variability and on the effect of the microbiome on plant performance in oilseed rape together with the lack of simple and cost-efficient high-throughput phenotyping methods make breeding for a modified microbiome for insect resistance a breeding target with a long-term perspective (Table 2, Fig. 1).

## Conclusion

Ensuring environmentally sustainable rapeseed production despite increasing threats from insect predation is a major challenge. Not only is rapeseed the target of approximately 40 species of insects worldwide, but very little resistance against insect predation has been so far identified in this species, and few effective control methods have been established. Phenotyping for resistance is also challenging; resistances are generally quantitative, and use of the few known chemical treatments or sprays is strictly regulated. In this review, we comprehensively outline different strategies for targeted research and breeding which may allow continued rapeseed production and propose an integrated strategy for future rapeseed crop protection against insect pests. Specifically, we highlight possible methods to identify (automated and high-throughput phenotyping, screening for secondary metabolite production) and transfer resistances (interspecific hybridisation, genetic engineering), suggest breeding targets (glucosinolates, volatiles and production of other secondary metabolites, microbiome features, plant surface barriers) and propose management strategies (identification of biological control methods such as insect predators and novel insect repellent compounds). Due to the high diversity of pest insects, no single compound or compound class will be a suitable breeding target for creating oilseed rape lines with multiple insect species resistances. By targeting a range of possible insect control methods, we hope that new, integrated protection strategies for rapeseed will be developed over the short, medium and long term, sustaining production of this major oil crop species.
